# A Panel of Urinary Long Non-coding RNAs Differentiate Bladder Cancer from Urocystitis

**DOI:** 10.7150/jca.37006

**Published:** 2020-01-01

**Authors:** Xiao Yu, Ruiwei Wang, Chenglin Han, Zilong Wang, Xunbo Jin

**Affiliations:** 1 Department of Urology, Shandong Provincial Hospital Affiliated to Shandong University, No. 324 Jingwu Road, Huaiyin District, Jinan, Shandong, 250021, China.; 2 Department of Anesthesiology, Shandong Provincial Hospital Affiliated to Shandong University, No. 324 Jingwu Road, Huaiyin District, Jinan, Shandong, 250021, China.

**Keywords:** Biomarker, NMIBC, urocystitis, non-coding RNA, expression.

## Abstract

Liquid biopsy is becoming a promising method for non-invasive cancer detection. In several proof-of-concept studies, long non-coding RNAs (lncRNAs) were found to be potential biomarkers for bladder cancer detection. The objective of this study was to discover a panel of cell-free, urinary lncRNAs as liquid biopsy biomarkers to non-invasively differentiate bladder cancer from chronic urocystitis. To this end, we collected urine samples from both bladder cancer patients and urocystitis patients. These samples were divided into discovery group and validation group. In the discovery group, the expression levels of 16 cell-free urinary lncRNAs were measured by qPCR to discover candidate biomarkers. The diagnostic performance of the candidate lncRNA biomarkers was then evaluated, which led to a panel of lncRNA biomarkers for bladder cancer detection. The performance of this panel of biomarkers was further evaluated in the validation group to see if these lncRNA biomarkers could discriminate the bladder cancer patients from urocystitis patients. We found that all of the 16 lncRNAs evaluated in this study demonstrated significant difference (*p*<0.05) of expression between bladder cancer patients and urocystitis patients. Nine lncRNAs provided decent diagnostic performance with area under the receiver operating characteristic (ROC) curve (AUC) reaching 0.70 or higher. We then selected the top four lncRNAs, namely UCA1-201, HOTAIR, HYMA1 and MALAT1, to form a panel of urinary biomarkers. Using this panel, bladder cancer patients could be discriminated from urocystitis patients, with sensitivity and specificity reaching 95.7% and 94.3%, respectively. Finally, we confirmed the applicability of the four-lncRNA panel in an independent validation study that included 60 bladder cancer patients and 60 urocystitis patients. Our study paves the way for further studies aimed at large-scale clinical tests of developing lncRNA biomarkers in urine for bladder cancer diagnostics.

## Introduction

Malignant bladder carcinomas are the most common tumors in urinary systems. Bladder cancer concerns approximately 549,393 new cases each year worldwide and its incidence is constantly increasing 1, 2. At time of diagnosis, non-muscle invasive bladder cancer (NMIBC) represents the majority of cases, accounting for 70% 1-9. Early diagnosis and treatment of cancerous or precancerous lesions is thought to be important for reducing the risk of relapse and improving the prognosis of NMIBC 10. However, the differentiation of chronic urocystitis from NMIBC can be particularly difficult, especially after previous treatment with attenuated mycobacteria (Bacillus Calmette-Guèrin) for deliberate induction of an inflammatory reaction 6, 8. This treatment is often a primary treatment option for NMIBC aside from early cystectomy 6, 8. Therefore, correct discrimination of urocystitis from NMIBC calls for a panel of biomarkers with both high sensitivity and high specificity.

Recently, several reports have highlighted the role of long non-coding RNAs (lncRNAs) as urine-based biomarkers for diagnosis of bladder cancer 1, 10-22. Long non-coding RNAs are transcripts longer than 200 nucleotides that are not translated into protein 23. LncRNAs do not contain open reading frames, but have conservative secondary structures. LncRNAs could interact with DNA, RNA or proteins as molecular sponges, scaffolds and activators to play important regulatory roles in a variety of biological processes 11. For example, Wang and collaborators 13 determined that high expression of UCA1 (Urothelial cancer associated 1) in urine sediments allows detection of high-grade superficial bladder tumors. Another study 14 showed that overexpression of several lncRNAs, such as HOTAIR, HOX-AS-2, MALAT1, HYMAI, LINC00477, LOC100506688 and OTX2-AS1, has been found in urine exosomes of high-grade MIBC patients. Several other studies 10, 12 also proposed various panels of lncRNAs as liquid biopsy biomarkers to discriminate bladder cancer patients from healthy controls.

Encouraged by these successes, in this study, we attempted to discover and evaluate the prediction power of a panel of lncRNAs biomarkers to discriminate bladder cancer from urocystitis. We chose totally sixteen lncRNAs as our candidate biomarkers, as suggested by previous studies 1, 10-22. We conducted a study of 140 NMIBC patients and 140 urocystitis patients. We found that the expression profiles of sixteen cell-free lncRNAs evaluated in urine were significantly different between NMIBC patients and urocystitis patients. Among these biomarkers, we chose four lncRNAs, namely UCA1-201, HOTAIR, HYMA1 and MALAT1, as our panel of lncRNA biomarkers for further investigation, due to their high value of area under curve (AUC) of the receiver-operating characteristic (ROC) curve. We found that this panel discriminated the NMIBC patients from urocystitis patients with high sensitivity (95.7%) and high specificity (94.3%). To further evaluate the clinical performance of our method, we then applied this cell-free, urinary lncRNA panel to an independent clinic study that included 60 NMIBC patients and 60 urocystitis patients. The specificity of our method remained as high as 96.7%: only 2 out of 60 urocystitis patients scored as cancer positive. The sensitivity of our method was 93.3%: 56 out of 60 cancer patients scored as cancer positive. These studies confirmed that the lncRNA panel could serve as a robust, accurate, and non-invasive method for bladder cancer detection. To our best knowledge, our study represents the first attempt of using cell-free urinary lncRNA biomarkers for non-invasive differentiation of bladder cancer from urocystitis.

## Materials and methods

### Patient populations and ethnics

We were approved for this study by the Institutional Review Board of Shandong Provincial Hospital Affiliated to Shandong University (Jinan, China) and conducted this study in accordance with the principles of Declaration of Helsinki. For all research participants, we obtained written-informed consent forms. In the cohort for biomarker discovery phase (Table 1), a total of 140 NMIBC patients, and 140 people that were diagnosed as severe urocystitis with reactive urothelial atypia (RUA) were recruited in the period of April 19^th^, 2015 and January 17^th^, 2018. The following criteria were used to include or exclude research participants: 1) inclusion criteria: confirmed bladder cancer that was diagnosed via cytologic examination; and 2) exclusion criteria: NMIBC patients that had chemotherapy/radiotherapy within 1 month, or NMIBC patients that were diagnosed of other malignancies within five years prior to urine collection.

### Urine sample collection and measurement of lncRNA expression levels in urine

Urine samples were collected on the day before treatment and kept on ice during collection. To remove cells and debris, urine samples were centrifuged at 1,500 g for 10 minutes and 13,800 g for 15 minutes at 4^o^C by following a previously published protocol 10. We followed a protocol that was previously published for extracting cell-free, urinary lncRNA 10. In brief, total RNA was isolated from 400 μL urine by using the miRNeasy Mini Kit (Qiagen) and following the manufacturer's instructions. We used NanoDrop spectrophotometer to measure the concentration of isolated RNA. Reverse transcription of urinary lncRNA was conducted by using the PrimeScript™ RT reagent kit (Takara, Dalian, Liaoning), in which the 20 µL RT volume contained 1 μg of template RNA, 4 μL of 5 × PrimeScript Buffer, 1 μL of PrimeScript RT Enzyme Mix I, 1 μL of Oligo dT Primer and RNase - free dH2O. The mixture was briefly centrifuged and incubated at 37°C for 30 minutes, followed by incubation at 85°C for 5 seconds and 4°C for 60 minutes. The quantitative polymerase chain reaction was conducted in a 25 μL reaction system, which contained 12.5 μL of SYBR® Premix Ex Taq™, 0.5 μL of ROX Reference Dye α, 1 μL of forward primer (10 μmol/L), 1 μL of reverse primer, 8 μL of RNas - free dH2O and 2 μL of cDNA on a CFX-96 real-time PCR System using the SYBR® Premix Ex Taq™ (Takara, Dalian, Liaoning). All reactions were performed in triplicate. GAPDH was used as an internal control. Raw Ct data was normalized by subtracting GAPDH Ct values from the biomarker Ct values to generate ΔCt. The relative gene expression were calculated by following the 2^-ΔCT^ method as reported previously 24, where relative gene expression in a urine sample = 2 ^ (- ΔCt) and multiplying by 1000.

### Statistical methods and machine learning analysis

Boxplot of relative lncRNA expression level was conducted by using MedCalc software (MedCalc Software bv, Ostend, Belgium). Two-sided p-value was calculated when comparing the expression level between patient group and control group. A p-value smaller than 0.05 was chosen as statistically significant. For each biomarker, we performed receiver operating characteristic (ROC) analysis, followed by calculating the area under the curve (AUC) using MedCalc software. Based on the AUC value of each biomarker, we could decide whether or not a biomarker was discriminative in separating NMIBC patients and urocystitis patients. We chose the biomarkers that had AUC values larger than 0.70 to develop a predictive model. We selected decision tree algorithm as our classifier and applied 10-fold cross-validations to avoid overfitting. The classification was conducted by using the data collected in the biomarker discovery phase. Python scikit-learn package was used for the computational analysis. The classifier was then used for the data collected in the validation phase to predict whether or not a sample was from NMIBC patients.

## Results

### Study design

We designed our study by including a biomarker discovery phase and an independent clinical validation phase. The discovery phase aimed at designing a panel of cell-free urinary lncRNA biomarkers to differentiate NMIBC patients from urocystitis patients. The validation phase aimed at using this panel in clinical diagnosis to evaluate its performance of NMIBC detection. In discovery phase, we recruited 140 NMIBC patients and 140 urocystitis patients (Table 1) and collected urine samples to measure the cell-free expression level of sixteen candidate lncRNAs. These data formed the training datasets, on which we applied a machine learning model and 10-fold cross-validation to determine the panel's sensitivity and specificity. Next, using the developed multi-lncRNA panel, we performed a blinded test to predict if a urine sample was from a NMIBC patient or urocystitis patient. We recruited a total of 60 NMIBC patients and 60 urocystitis patients in the validation phase.

### Evaluating expression profiles of candidate lncRNA biomarkers in urine for NMIBC detection

**Figure 1:**
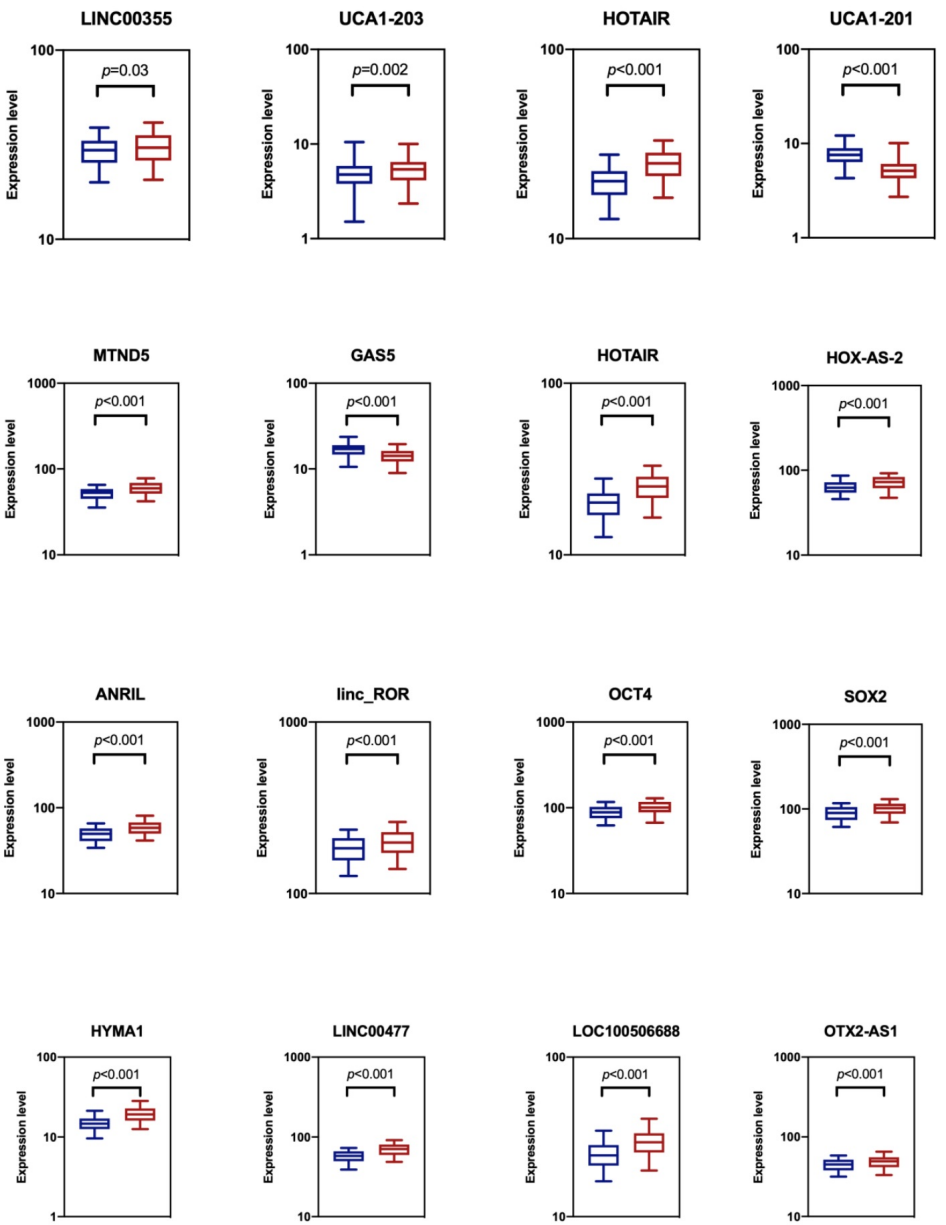
Comparison of cell-free expression levels of sixteen candidate lncRNA biomarkers in urine between NMIBC group (blue) and urocystitis group (red). The *p*-value from t-test analysis was calculated for each comparison. We considered *p*<0.05 as statistically significant.

We analyzed the expression levels of totally sixteen candidate biomarkers in two groups of the training datasets: a NMIBC group and urocystitis group (Figure 1). The NMIBC cancer group consisted of 140 NMIBC patients, while the urocystitis group consisted of 140 patients diagnosed with severe urocystitis with RUA. These candidate lncRNA biomarkers were selected because they have been previously reported to be able to discriminate bladder cancer patients from healthy controls 10, 12, 14. We compared the extracellular expression levels of each candidate biomarker in urine between NMIBC group and urocystitis group. We found that all of the candidate biomarkers demonstrated elevated expression levels in the NMIBC group except for UCA1-201 and GAS5, with HYMA1 showing the largest up-regulation (1.31-fold). UCA1-201 and GAS5, on the other hand, demonstrated decreased expression level in the NMIBC group (1.48-fold and 1.18-fold, respectively). The differentiated expressions were significant (*p*<0.05) for all candidate biomarkers, indicating the feasibility of using these biomarkers for potential classification of bladder cancer.

### Discovering a panel of cell-free urinary lncRNA biomarkers

**Figure 2:**
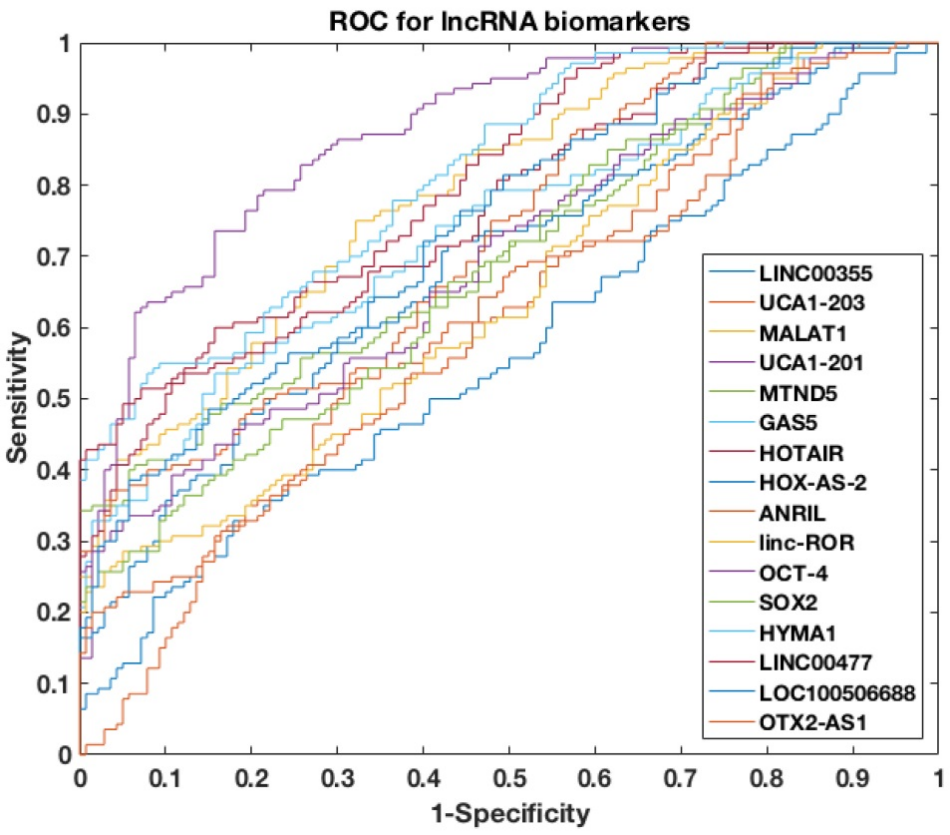
ROC curves of single lncRNA biomarker used to differentiate NMIBC and urocystitis in the training datasets.

To further assess the power of differentiating NMIBC patients from urocystitis patients, the receiver operating characteristic (ROC) curve was evaluated for each candidate biomarker, followed by area under curve (AUC) calculation (Figure 2). Among the sixteen candidate biomarkers, nine biomarkers (i.e., UCA1-201, HOTAIR, HYMA1, MALAT1, LINC00477, LOC100506688, GAS5, ANRIL, and MTND5) demonstrated AUC values higher than 0.70 (i.e., an AUC value that normally suggests decent separation of clinical positives from negatives), while the other seven biomarkers had AUC values below 0.70. The AUC of top four biomarkers, i.e., UCA1-201, HOTAIR, HYMA1 and MALAT1, were even higher than 0.80, suggesting superior diagnostic performance in differentiating NMIBC and urocystitis.

To further explore the potential of using cell-free urinary lncRNAs as biomarkers to differentiate NMIBC and urocystitis, we then constructed a four-lncRNA panel that included UCA1-201, HOTAIR, HYMA1 and MALAT1. We applied a machine-learning model to predict NMIBC occurrence using the four-lncRNA panel. The input of this predictive model was the cell-free expression levels of the four lncRNAs, paired with the phenotype data (i.e., NMIBC or urocystitis). Totally 280 datasets were retrieved in the training phase, which was then used to train the model. Decision-tree algorithm was chosen as the classifier and python scikit-learn package was adopted for computation, which automatically adjusted the nodes and connections of the decision tree to optimize the fitting 25. The predictive dramatically improved the discriminative power, as the AUC of the ROC curve reached 0.95.

### Validating biomarker panel with independent datasets

**Figure 3:**
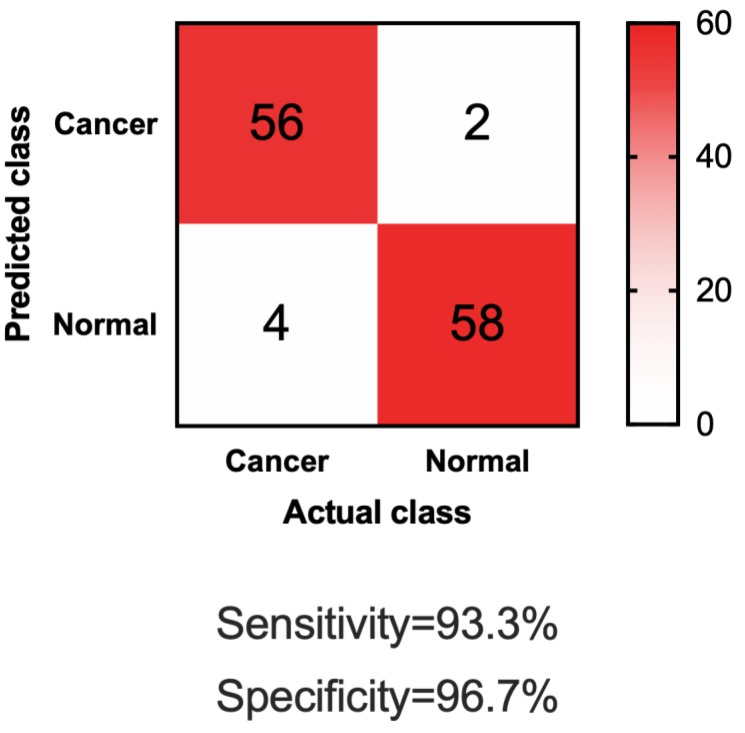
Confusion matrix of applying the four-lncRNA panel to differentiate NMIBC and urocystitis in the validation datasets.

To further test if our four-lncRNA panel could be generally applied for differentiating NMIBC patients from urocystitis patients, we sought validation in independent datasets. In this phase of independent biomarker panel validation, we conducted a study of 60 NMIBC patients and 60 urocystitis patients, blinded. As shown in Table 1, demographic information of the participants in the validation phase was similar to those in the discovery phase. The urine samples were obtained from participants in the validation phase, blinded, and analyzed for the cell-free expression level of UCA1-201, HOTAIR, HYMA1 and MALAT1. Compared to samples collected from NMIBC patients and urocystitis patients in the discovery phase, none of the lncRNA expression showed significant difference (*p*>0.05) in the validation phase. Using the machine learning guided, predictive model trained in the discovery phase, we made predictions on the samples in the validation phase (i.e., whether or not one sample was from a NMIBC patient). Totally 58 out of 60 urocystitis patients scored negative, and 56 of 60 NMIBC patients scored positive (Figure 3). The sensitivity reached 93.3% and the specificity 96.7%. The high sensitivity and specificity supported the application of the four-lncRNA panel in urine samples as a potentially non-invasive approach for clinical diganosis for NMIBC.

## Discussion

Our discovery of a panel of four lncRNA biomarkers in urine (i.e., UCA1-201, HOTAIR, HYMA1 and MALAT1) presents a promising and unique means for differentiating NMIBC patients from urocystitis patients. Using this panel, we were able to achieve high specificity and high sensitivity in both training datasets and independent validation datasets. The candidate lncRNA biomarkers have been evaluated on differentiating bladder cancer patients from healthy controls 10, 12, 14. Our study complements previous efforts and expand the usage of liquid biopsy lncRNA biomarkers for discriminating bladder cancer patients from inflammatory patients. To our best knowledge, it is the first time that cell-free urinary lncRNA biomarkers were used to non-invasively separate bladder cancer patients and urocystitis patients.

One of the novel findings from this study is that certain lncRNA biomarkers, which were used to detect bladder tumors from healthy controls, did not perform well in separating bladder tumors from urocystitis. In this study, we evaluated sixteen lncRNA biomarkers, all of which were found to be effective in discriminating bladder tumors from healthy controls 10, 12-14. However, among the sixteen biomarkers, seven of them, namely LINC00355, UCA1-203, HOX-AS-2, Linc-ROR, OCT4, SOX2 and OTX2-AS1, did not demonstrate sufficient discriminatory power to separate bladder tumors from urocystitis, as indicated by their low AUC values (AUC<0.70). While investigating the biomolecular mechanism of lncRNA biomarkers is beyond the scope of this study, we do want to raise the hypothesis that during the development of urocystitis, the transcriptome of urine was altered by inflammation, which could potentially cause the reduced effectiveness of certain biomarkers. Our finding also highlights the necessity of developing “cancer vs. inflammation” biomarkers to specifically discriminate bladder tumors from urocystitis in clinical applications.

We also would like to pinpoint a few limitations of this study. As mentioned in the paragraph above, in this study we aimed at discovering and validating biomarkers of bladder tumors, but not focused on uncovering the molecular insights into the mechanisms of cell-free lncRNA expression. We do want to point out that, as suggested by Raposo et al. 26, extracellular vesicles, such as exosomes, microvesicles and apoptotic bodies, can contribute to the remarkable stability of cell-free lncRNAs. We will design further studies to analyze the origin and protection mechanisms of cell-free lncRNAs in urine samples. Also, our study represented a single-center research. Larger number of independent cohorts from multi-centers are needed to validate our current findings. Finally, we will continue to improve the specificity of the constructed urinary lncRNA panel for bladder cancer diagnosis, especially for early-stage patients. We will also need to evaluate the discriminatory power of our lncRNA panel between bladder cancer patients and other patients with non-cancerous diseases (e.g., urinary tract infection).

In summary, we concluded that a panel of four lncRNA biomarkers could be used to differentiate bladder cancer from urocystitis, with high sensitivity and high specificity. This pave the way for further predictive study followed by pivotal clinical validation.

## Figures and Tables

**Table 1:** Demographic Information of All Subjects Used in This Study.

Groups	Training datasets	Validation datasets	*P*-Value
Control Group	Patients recruited in training phase	Patients recruited in validation phase	
	140 urocystitis patients	60 urocystitis patients	
	Age (years)	Age (years)	0.58
	<66: 74 (52.85%)	<66: 29 (48.33%)	
	≥66: 66 (47.15%)	≥66: 31 (51.67%)	
	Cigarette smokers	Cigarette smokers	0.32
	37 (26.43%)	17 (28.3%)	
	Alcohol drinkers	Alcohol drinkers	0.24
	19 (13.57%)	8 (13.33%)	
	Sex	Sex	0.34
	Male: 92 (65.71%)	Male: 39 (65.00%)	
	Female: 48 (34.29%)	Female: 21 (35.00%)	
	Condition	Condition	0.89
	Cystitis: 140 (100%)	Cystitis: 60 (100%)	
Bladder Cancer Group	Patients recruited in training phase	Patients recruited in validation phase	
	140 NMIBC patients	60 NMIBC patients	
	Age (years)	Age (years)	0.52
	<66: 72 (51.43%)	<66: 33 (55.00%)	
	≥66: 68 (48.57%)	≥66: 27 (45.00%)	
	Cigarette smokers	Cigarette smokers	0.31
	45 (32.14%)	19 (31.7%)	
	Alcohol drinkers	Alcohol drinkers	0.27
	23 (16.43%)	9 (15.00%)	
	Sex	Sex	0.93
	Male: 96 (68.57%)	Male: 41 (68.33%)	
	Female: 44 (31.43%)	Female: 19 (31.67%)	
	Tumor stage	Tumor stage	0.21
	Ta-T1: 78 (55.71%)	Ta-T1: 35 (58.33%)	
	T2-T4: 62 (44.29%)	T2-T4: 25 (41.67%)	

**Table 2:** Primers Used in This Study.

Primer name	Sequence
LINC00355_F	TGGGTCTCCTCTGAGCTGTT
LINC00355_R	TGTCCTGTGTCCAGGATGAA
UCA1-201_F	GCTTAGTGGCTGAAGACTGATG
UCA1-201_R	TCATATGGCTGGGAATCCTC
UCA1-203_F	GCATCCAGGACAACACAAAG
UCA1-203_R	ACCCTTTTCCCATAGGTGTG
MALAT1_F	CTTCCCTAGGGGATTTCAGG
MALAT1_R	GCCCACAGGAACAAGTCCTA
HOTAIR_F	TCCCCTACTGCAGGCTTCTA
HOTAIR_R	CCTAATATCCCGGAGGTGGCT
HOXA-AS2_F	GCTCTCTCCTGCCTTCCTG
HOXA-AS2_R	AGCTTGGCCTACTGTGGAAA
ANRIL_F	GCCTCATTCTGATTCAACAGC
ANRIL_R	GATCTCCCCGGTTTTCTTCT
Linc-RoR_F	CTGGCTTTCTGGTTTGACG
Linc-RoR_R	CAGGAGGTTACTGGACTTGGAG
OCT4_F	GAAAGCGAACCAGTATCGAGAAC
OCT4_R	CCCCTGAGAAAGGAGACCCA
SOX2_F	ACCAGCTCGCAGACCTACAT
SOX2_R	TGGAGTGGGAGGAAGAGGTA
HYMA1_F	TTGCCTTTGTTTTCCTCCAG
HYMA1_R	ACGCAATTGAATGGGAAAG
LINC00477_F	CTCCTCCTCCTTGGCCTACT
LINC00477_R	CAGCCTGGACAACAGAGTGA
LOC100506688_F	GTGGCTGAGAGGCTACAGGA
LOC100506688_R	GAGGTCTGTCGCTGGACTTT
OTX2-AS1_F	TGAAAGCGATGATGATGTCG
OTX2-AS1_R	GCAACAAGAGCCAGGTAAGG
MTND5_F	TCATAATAGTTACAATCGGCAT
MTND5_R	TAATGAGAAATCCTGCGAATA
GAS5_F	CTTCTGGGCTCAAGTGATCCT
GAS5_R	TTGTGCCATGAGACTCCATCAG
GAPDH_F	CTGGGCTACACTGAGCACC
GAPDH_R	AAGTGGTCGTTGAGGGCAATG

## Abbreviations

lncRNA: long non-coding RNA
ROC: receiver operating characteristic
AUC: area under curve
NMIBC: non-muscle invasive bladder cancer
UCA1: Urothelial cancer associated 1
RUA: reactive urothelial atypia
